# Cytoreductive surgery for ovarian cancer with pelvic ectopic kidney: a case report

**DOI:** 10.3389/fonc.2026.1815298

**Published:** 2026-05-15

**Authors:** Mingqin Kuang, Xiyun Cheng, Meiru Wen, Changmei Shen

**Affiliations:** 1 Department of Gynecology, Ganzhou Cancer Hospital, Ganzhou, China; 2 Department of Radiology, Ganzhou Cancer Hospital, Ganzhou, China

**Keywords:** cytoreductive surgery, MDT, multidisciplinary team, ovarian cancer, pelvic ectopic kidney

## Abstract

Pelvic ectopic kidney is a rare congenital anomaly, occurring in approximately 0.1% of the adult population, and is often asymptomatic. However, it can easily be mistaken for a pelvic tumor or lymph-node metastasis. This case report details a rare presentation of cytoreductive surgery for ovarian cancer with a pelvic ectopic kidney. A 56-year-old Chinese woman was incidentally found to have a pelvic mass accompanied by a pelvic ectopic kidney during evaluation for hip pain. Pathology following cervical lymph-node biopsy was consistent with high-grade serous adenocarcinoma. Comprehensive assessment via supraclavicular lymph-node biopsy, PET-CT, and multidisciplinary team (MDT) discussion confirmed ovarian cancer stage IVb with coexisting pelvic ectopic kidney. Treatment consisted of neoadjuvant chemotherapy with paclitaxel plus carboplatin followed by interval cytoreductive surgery achieving complete resection of all pelvic and abdominal lesions. Postoperative pathology verified high-grade serous ovarian carcinoma, stage IVb. Serum creatinine on postoperative day 1 was 78 µmol/L and remained stable; the patient was discharged on day 12. Post-discharge renal scintigraphy showed no decline in total GFR, and no renal or surgical complications occurred. This case underscores the importance of MDT management and thorough diagnostic work-up when performing cytoreductive surgery for ovarian cancer in the setting of pelvic ectopic kidney. Increased clinical awareness of this scenario helps avoid misdiagnosis, optimizes patient outcomes, and provides a reproducible technical roadmap for similar complex cases.

## Introduction

Ovarian cancer remains the most lethal gynecologic malignancy, with a high mortality rate largely attributed to its insidious onset and the absence of specific early symptoms (1). Consequently, approximately 70% of patients are diagnosed at an advanced stage, where the disease has already spread beyond the ovaries to involve the peritoneum or distant organs (2). The cornerstone of treatment for advanced ovarian cancer is maximal cytoreductive surgery, with the goal of achieving no visible residual disease (R0 resection) (3). This has been consistently identified as an independent prognostic factor for improved survival (4). The surgical approach has evolved to include more extensive procedures, such as peritonectomy, lymphadenectomy, and bowel resection, to achieve complete tumor removal (5). However, this aggressive surgical strategy also increases the complexity of the procedure and the potential for complications, particularly in the presence of anatomical variations or anomalies.

A pelvic ectopic kidney is a rare congenital anomaly resulting from the failure of the kidney to ascend from the pelvis to its normal retroperitoneal position during embryonic development (6, 7). This condition is present in approximately 1 in 2, 100 to 1 in 3, 000 individuals and is often asymptomatic (8, 9). The common diagnostic methods for pelvic ectopic kidney mainly rely on the combination of B-mode ultrasound and three-dimensional computed tomography (3D-CT) (10). However, its presence can be a significant source of diagnostic confusion and surgical risk. The kidney’s location in the true pelvis, often in close proximity to the uterus, adnexa, and colon, makes it vulnerable to injury during gynecological procedures (11, 12). There are two main reasons for the confusion between pelvic ectopic kidney and pelvic masses:First, ectopic kidneys are prone to locate in the pelvic cavity due to gravitational effects or congenital developmental abnormalities, which can be confused with organs that normally reside in the pelvis, such as the ovaries and uterus.Second, ectopic kidneys typically have their own vascular supply, and the hilar vessels are easily confused with the ovarian vessels and infundibulopelvic ligament (13). On imaging, the primary differential diagnostic method is three-dimensional computed tomography angiography (3D-CTA). This requires close collaboration between clinicians and radiologists to carefully analyze the blood supply origin of the pelvic mass, thereby further distinguishing between an ectopic kidney and a pelvic tumor (14). We reviewed previous literature and found no reports of ovarian cancer complicated with pelvic ectopic kidney. However, in the field of gynecology, there has been a case report of an undeveloped uterine horn combined with ovarian endometriosis presenting as a pelvic ectopic kidney. In that case, the patient underwent laparoscopic resection of the left pelvic ectopic kidney due to the non-functioning nature of the left kidne (15). The vascular supply of a pelvic ectopic kidney is highly variable and anomalous, with arteries often originating from the iliac vessels or the aorta, and veins draining into the iliac veins or the inferior vena cava (16). Ovarian cancer cytoreductive surgery complicated with pelvic ectopic kidney presents numerous challenges. The pelvic ectopic kidney occupies the central pelvic region and is prone to adhesion with ovarian tumors or metastatic lesions, which obscures the surgical field and increases the risks of residual tumor and accidental resection of renal parenchyma. Its blood supply originates from variant branches of the iliac vessels or the lower segment of the abdominal aorta, with tortuous anatomical courses. Injury to these vessels is likely during pelvic lymphadenectomy or pelvic vascular manipulation, leading to massive hemorrhage. The ureter may have an abnormal course and close anatomical proximity to the tumor and pelvic organs. Accidental resection or perforation of the ureter can occur during dissection or resection of invaded tissues, resulting in urinary fistula or hydronephrosis. Congenital adhesions combined with tumor infiltration form dense adhesive masses, which increases the difficulty of dissection and raises the risk of injury to the renal parenchyma or ureter. Ectopic kidneys are often associated with potential renal insufficiency. When the kidney is invaded by the tumor, clinicians must balance the extent of cytoreduction and renal function protection, facing the dual challenges of residual tumor and renal function impairment (17). For these potential risks, preoperative multidisciplinary team (MDT) discussion is particularly crucial. When confronted with anatomical structures unfamiliar to one’s own specialty, MDT serves not only as an excellent learning opportunity but also as practical surgical guidance.

Despite the known challenges associated with pelvic ectopic kidneys in abdominal and pelvic surgery, there is a paucity of literature specifically addressing the management of advanced ovarian cancer with cytoreductive surgery in patients with this anatomical variant. To the best of our knowledge, there have been no previous reports detailing a successful cytoreductive surgery for ovarian cancer in a patient with a pelvic ectopic kidney. This case report aims to fill this gap by presenting a detailed account of our experience, from the initial diagnostic challenges to the successful surgical outcome. By systematically outlining the key perioperative strategies, including the critical role of a multidisciplinary team and advanced imaging, we hope to raise awareness among gynecologic oncologists about this rare but important anatomical variant. The objective is to provide a practical framework for managing similar complex cases, with the ultimate goal of preventing preventable iatrogenic renal loss and optimizing patient outcomes.

## Case description

A 56-year-old Chinese female, 145 cm tall and 71.5 kg in weight, had a BMI of 34.01 kg·m^-^². In January 2025, she presented to a local hospital with right hip soreness. MRI accidentally revealed “left renal agenesis and right paracolic gutter soft tissue mass, “ without further management. In April 2025, she revisited the same hospital due to severe abdominal distension and underwent laparoscopic exploration and abdominal wall mass resection under general anesthesia. Postoperative pathology confirmed moderately-poorly differentiated adenocarcinoma, consistent with ovarian origin. As the patient initially visited a county hospital with relatively limited medical resources and expertise, the physicians there were not fully confident in managing this uncommon clinical condition. Therefore, the hospital advised the patient to receive treatment at a higher-level hospital. After consideration, the patient chose our hospital. She was transferred to our hospital for further treatment in June 2025. PET-CT showed metastases to the bilateral lungs, left supraclavicular lymph nodes, para-aortic lymph nodes, and right external iliac lymph nodes, with extensive peritoneal carcinomatosis, suggesting stage IVb ovarian cancer. The left kidney was identified in the pelvis, consistent with a pelvic ectopic kidney. Cervical lymph node biopsy confirmed adenocarcinoma of ovarian origin. She had no previous urinary symptoms, hypertension, or diabetes, and no family history of hereditary tumors.

To better illustrate the variant anatomy of the kidney, we have added a cartoon diagram. In a normal human, the left renal artery usually arises from the lateral wall of the abdominal aorta, whereas the blood collected by the left renal vein drains into the inferior vena cava (**Supplementary Figure 1A**). In contrast, a pelvic ectopic kidney may exhibit aberrant vascular courses; for example, the left renal artery may originate from the left common iliac artery, and both the left renal vein and the left ureter can display anatomical variations that differ from the standard renal architecture (**Supplementary Figure 1B**).

**Figure 1:**
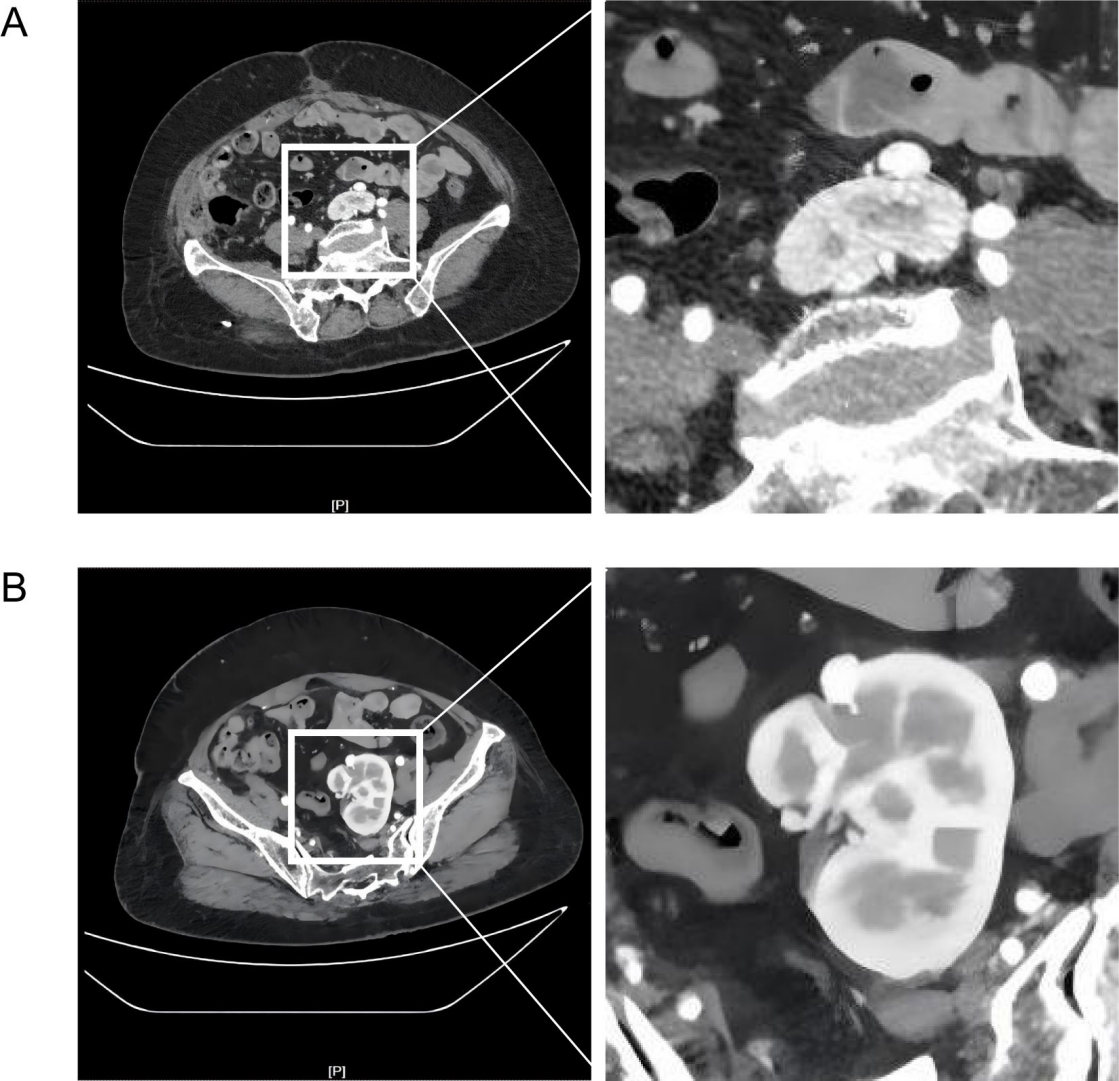
Preoperative contrast-enhanced CT localization of the pelvic ectopic kidney. **(A)** The upper pole of the left pelvic ectopic kidney is situated at the bifurcation of the left internal and external iliac arteries. **(B)** The overall anatomical position of the left pelvic ectopic kidney is at the level of the sacral promontory.

**Figure 2:**
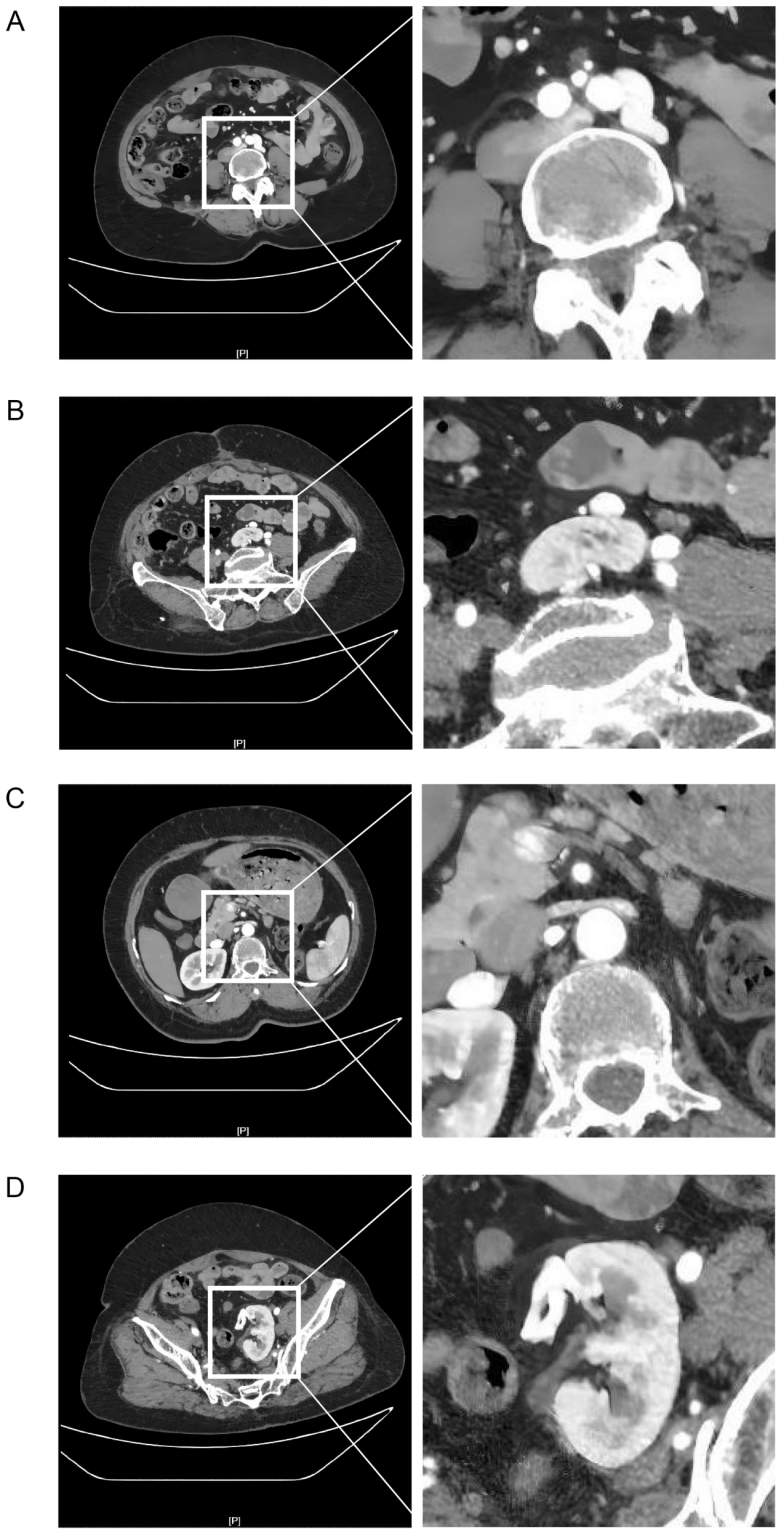
Variations in vascular anatomy of the left pelvic ectopic kidney on contrast-enhanced CT. **(A)** The left renal artery aberrantly originates from the left common iliac artery. **(B)** The left renal artery enters the renal hilum via the upper pole of the kidney. **(C)** The left renal vein drains into the inferior vena cava at a normal anatomical position. **(D)** The left renal vein divides into multiple branches at the renal hilum for venous drainage.

First, adequate imaging evaluation was performed. 1) Contrast-enhanced CT: The upper pole of the left kidney was located at the bifurcation of the left internal and external iliac arteries (**Figure 1A**), and the entire left kidney was at the sacral promontory level (**Figure 1B**). The left renal vessels showed anatomical variation: the left renal artery originated from the left common iliac artery (**Figure 2A**) and entered the left renal hilum along the upper pole (**Figure 2B**). The left renal vein drained into the inferior vena cava at a normal location (**Figure 2C**) and divided into multiple branches at the renal hilum to collect venous blood (**Figure 2D**). Meanwhile, imaging examination revealed no obvious tumor infiltration around the left kidney (**Supplementary Figure 2**). 2) Renal dynamic imaging: The glomerular filtration rate (GFR) of the affected kidney was 42 mL·min^-^¹, accounting for 48% of total GFR, indicating good renal function. 3) CT three-dimensional reconstruction: The lower pole of the left kidney was adherent to the left adnexa and sigmoid mesocolon with blurred boundaries, easily misidentified as a metastatic lesion.

The initial imaging report identified the pelvic round soft tissue mass as the lower pole of the kidney displaced by an ovarian tumor. During the discussion of MDT, re-evaluation by urologists and radiologists confirmed continuity between the mass and renal parenchyma, with consistent enhancement timing to the kidney. Multiplanar reconstruction showed an anteriorly located renal hilum. The final diagnosis was revised to pelvic ectopic kidney complicated with stage IVb ovarian cancer, and a treatment strategy of NACT followed by interval cytoreductive surgery was formulated.

**Figure 3:**
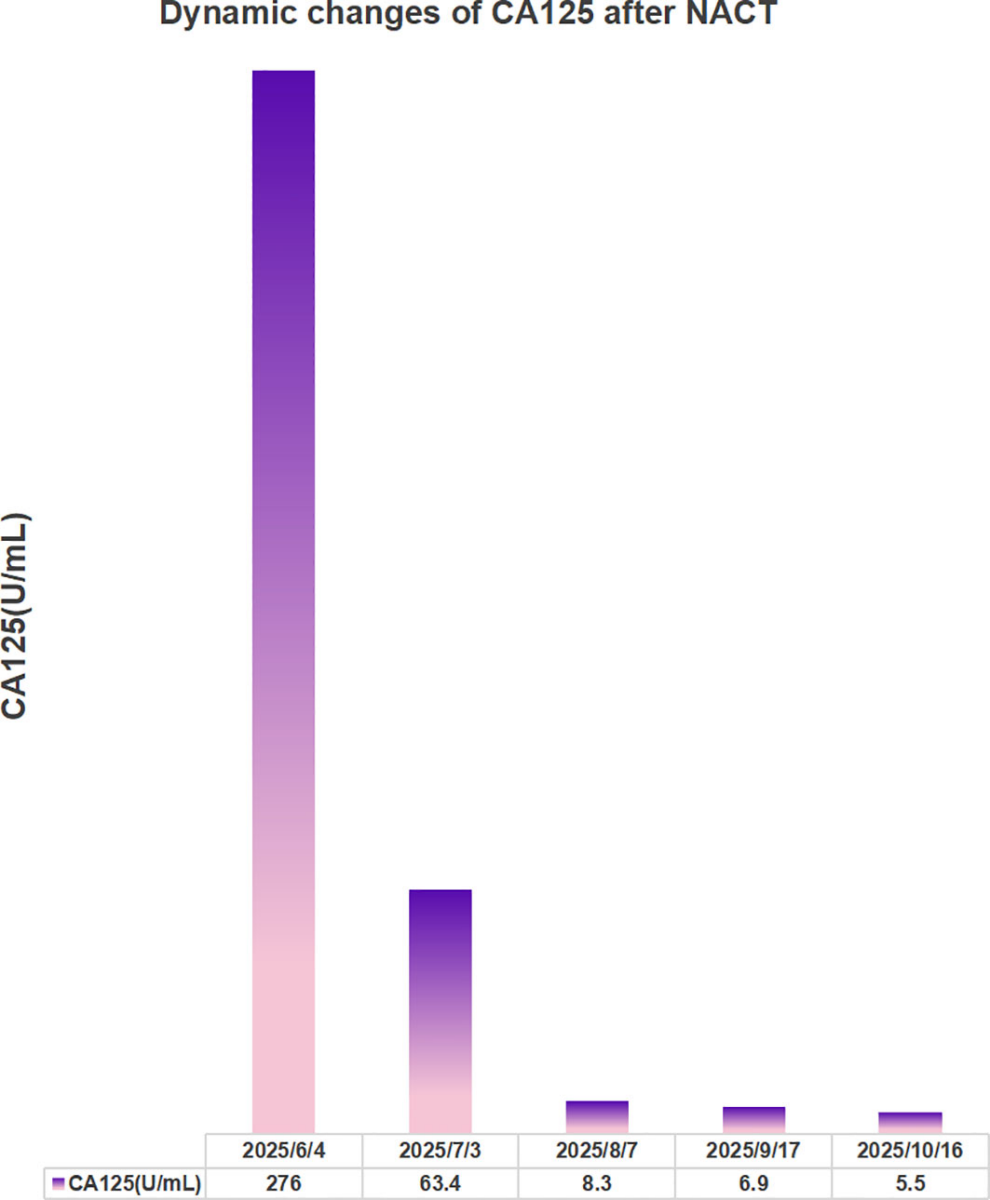
Dynamic changes in serum CA125 levels during neoadjuvant chemotherapy. Serum CA125 concentration decreased significantly from 276 U/mL to 5.5 U/mL after four cycles of paclitaxel plus carboplatin neoadjuvant chemotherapy, indicating a favorable chemotherapeutic response.

From June to September 2025, the patient completed 4 cycles of NACT with paclitaxel (175 mg·m^-^²) plus carboplatin (AUC 5). CA125 decreased from 276 U/mL to 5.5 U/mL (**Figure 3**). Follow-up CT showed a >50% reduction in peritoneal metastases, and left supraclavicular lymph node size decreased from 12 mm to 8 mm, indicating a satisfactory chemotherapeutic response. Meanwhile, the patient’s renal function remained within the normal range throughout the period of NACT (**Supplementary Figure 3**).

Urologists assessed that although the left renal pedicle was short, preoperative imaging showed no left pelvic lymphadenopathy. If left pelvic lymphadenectomy is not performed, the surgery could be performed without entering the retroperitoneum, minimizing renal injury risk. Intraoperative temporary suspension was planned if necessary. Based on current research evidence indicating that pelvic lymphadenectomy does not alter the prognosis of patients with advanced ovarian cancer, the gynecologic oncology team determined that it was unnecessary to incise the peritoneum covering the pelvic ectopic kidney or perform left pelvic lymphadenectomy during surgery. According to relevant treatment guidelines for ovarian cancer, IDS combined with NACT remains the optimal treatment regimen even for patients with advanced ovarian cancer (18). Anesthesiologists recommended preparing 4 units of blood and closely monitoring blood pressure to avoid fluctuations.

**Figure 4:**
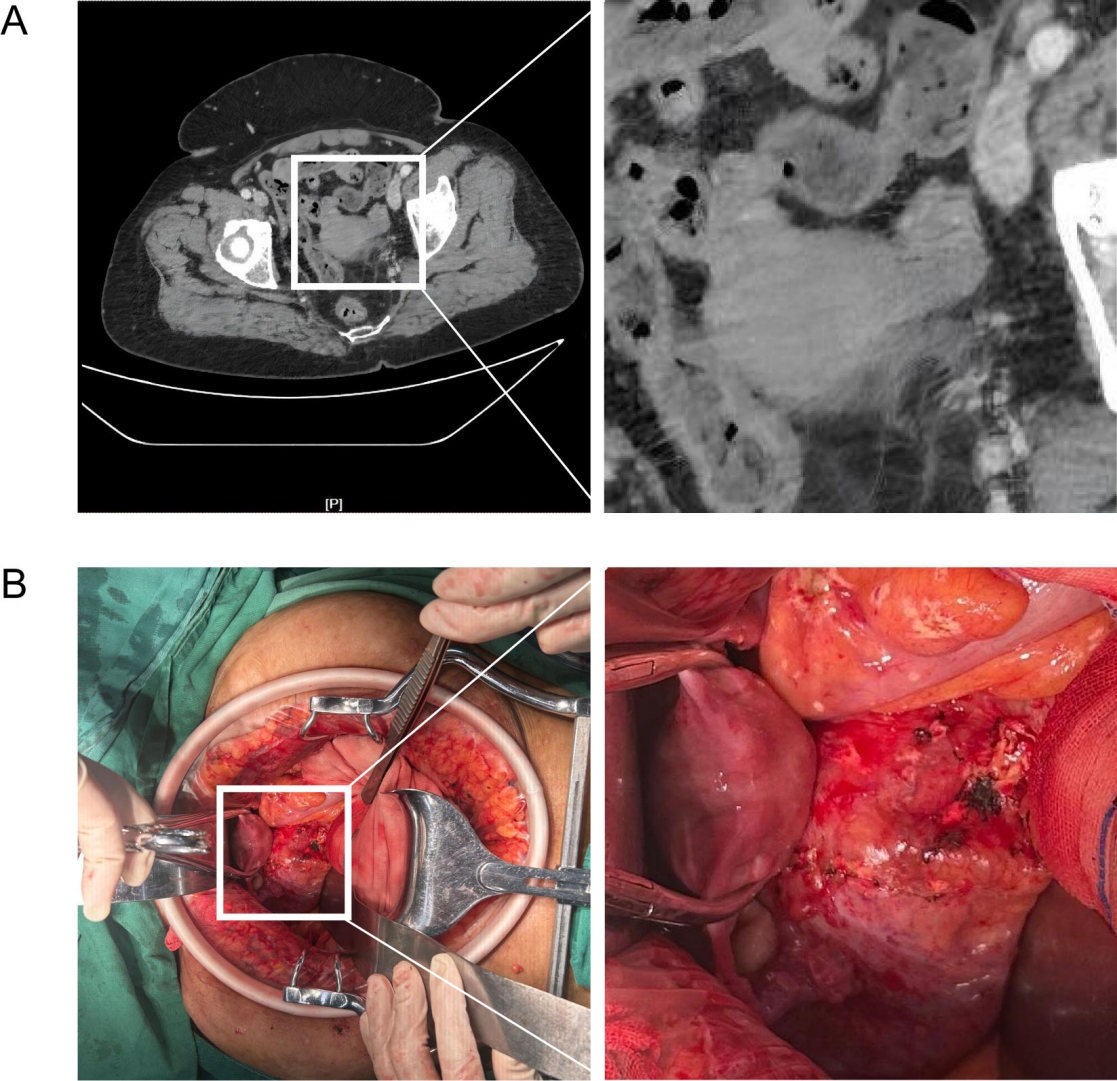
Anatomical correlation of the left pelvic ectopic kidney with surrounding pelvic structures. **(A)** Preoperative CT imaging demonstrates the intimate anatomical relationship between the left adnexa and the left pelvic ectopic kidney. **(B)** Intraoperative exploration shows the left pelvic ectopic kidney located anterior to the sacral promontory, with adhesion to the sigmoid mesocolon and the posterior uterine wall.

The operation is carried out according to the following steps: 1) Incision: Midline laparotomy from xiphoid process to pubic symphysis, curving around the umbilicus. 2) Exploration: Preoperative CT suggested close proximity between the left adnexa and left kidney (**Figure 4A**). Intraoperatively, the left kidney was located anterior to the sacral promontory, adherent to the sigmoid mesocolon and posterior uterine wall (**Figure 4B**). 3) Tumor resection sequence: Uterus and bilateral adnexa → pelvic peritoneum → right paracolic gutter nodules → appendix → omental cake. 4) Irrigation: Intra-abdominal irrigation with 4000 mL of distilled water. The urology team assessed that there was no indication for ureteral stent implantation. No active bleeding was identified at surgical sites, and instruments were counted correctly. 5) Closure: Abdominal incision was closed with one abdominal drain and one subcutaneous drain placed.

The total duration of the operation was 4 hours and 36 minutes, with an estimated blood loss of approximately 320 mL. Postoperative pathology confirmed high-grade serous ovarian cancer with complete resection of all pelvic and abdominal lesions, 0/3 positive lymph nodes, and FIGO stage IVb. Postoperatively, renal dynamic imaging showed left renal GFR of 43 mL·min^-^¹ and right renal GFR of 44 mL·min^-^¹, with no change in total GFR. There is a close correlation between the patient’s stable postoperative GFR and the preoperative planning.

The patient had a subcutaneous fat layer thickness of approximately 6 cm. No fat liquefaction occurred due to the subcutaneous drain, which was removed on postoperative day 8. The incision healed by primary intention. There were no complications such as urinary leakage, renal vein thrombosis, or lymphocele.

Postoperatively, the patient experienced no dyspnea, flank pain, or other signs of thrombo-embolism. She expressed satisfaction with both the surgical plan and the healing of her incision, appreciating that cytoreduction for ovarian cancer had been achieved while preserving function of the left pelvic kidney. The patient received the same adjuvant treatment regimen postoperatively as that administered preoperatively. A total of 6 cycles of chemotherapy were performed, with one cycle every 21 days.Follow-up was conducted by the chronic disease manager of our department, mainly to monitor chemotherapy-related adverse reactions after discharge.Imaging evaluation and serum detection of CA125 and HE4 were performed upon the patient’s return for subsequent treatment.

## Discussion

The diagnosis of a pelvic ectopic kidney can be challenging, especially in the context of a gynecological malignancy (19, 20). The kidney’s abnormal location and its close proximity to the reproductive organs can lead to misinterpretation of imaging findings, as was initially the case in our patient (21). The ectopic kidney can be mistaken for a pelvic mass, a bulky lymph node, or an extension of the primary tumor, which can lead to inappropriate surgical planning and an increased risk of iatrogenic injury (22, 23). The use of advanced imaging techniques, such as 3D CT angiography, is crucial for accurately identifying the ectopic kidney and its vascular anatomy, thereby preventing such errors.

In addition to anatomical imaging, functional studies, such as renal dynamic scintigraphy, are essential for assessing the function of the ectopic kidney and guiding the surgical approach (24). While PET-CT can identify the ectopic kidney, it does not provide a quantitative assessment of its function (25). The combination of anatomical and functional imaging provides a comprehensive understanding of the patient’s unique anatomy and allows for a more informed and individualized surgical plan (26). This integrated approach is critical for achieving a successful outcome in these complex cases. In daily practice, pelvic ectopic kidney is still frequently labelled renal agenesis because the first-line imaging is often a low-resolution, non-contrast CT or MR study that cannot resolve the small, malrotated parenchyma or its aberrant vessels. The retro-vesical location and superimposed bowel gas further obscure the subtle renal outline, so the organ is simply not seen and reported as absent. The key imaging differential diagnostic features among pelvic ectopic kidney, pelvic metastatic lesions and other pelvic masses are summarized. Their typical CT and MRI manifestations, including renal collecting system, aberrant blood supply, and continuity of renal pelvis and ureter, provide evidence for accurate preoperative identification and misdiagnosis prevention. We therefore emphasise that any report of single kidney should trigger a targeted CT/MR angiogram or 3-D CT reconstruction; these techniques reliably reveal the ectopic parenchyma and its anomalous blood supply.

The primary goal of surgery in the case is to achieve a complete cytoreduction while preserving the function of the ectopic kidney. This requires a meticulous surgical technique and a thorough understanding of the anomalous anatomy. The key to avoiding iatrogenic injury is to clearly identify and protect the ectopic kidney and its vascular supply throughout the procedure. In our case, the use of a systematic approach and careful mobilization of the surrounding structures allowed for a safe and successful surgery.

The successful management of this complex case was heavily reliant on a robust MDT approach (27). The initial misinterpretation of the imaging was corrected only after a joint review by gynecologic oncologists, urologists, and radiologists. The urologist’s expertise was crucial in understanding the anatomical variant and planning the surgical strategy to protect the kidney. This collaboration ensured that all aspects of the patient’s complex condition were considered, leading to a safe and effective treatment plan.

One study reported a 70-year-old male whose computed tomography (CT) scan revealed a left pelvic ectopic kidney with 16-mm and 5-mm stones in the renal pelvis, accompanied by hydronephrosis. After six years of follow-up, the stones became impacted in the renal pelvis and hydronephrosis worsened, so open pyelolithotomy was performed (28). Another study described a young pregnant woman in her twenties who presented with severe abdominal pain and vaginal bleeding. Ultrasonography suggested a left tubal ectopic pregnancy and ipsilateral pelvic ectopic kidney. Laparoscopic left salpingectomy was performed, and the report concluded that laparoscopic treatment of tubal pregnancy can be safely performed in the presence of ipsilateral pelvic ectopic kidney (29). Additionally, a study noted that preoperative pelvic ultrasound failed to detect the uterus but revealed a cystic lesion in the right adnexa and an pelvic ectopic kidney in the pelvic center. After laparoscopic exploration, laparotomy was performed to enter the retroperitoneal space, and an 8×11 cm smooth-contoured perirenal cyst adjacent to the pelvic kidney was detected; postoperative pathology confirmed endometriosis (30). The distinguishing feature of our study is that the patient had aovarian cancer complicated with pelvic ectopic kidney. Our surgical resection scope was extensive, the operation time was longer, and the corresponding surgical challenges were greater. Fortunately, the patient’s left kidney maintained normal renal function, and no enlarged lymph nodes were found in the left pelvis, which reduced the risk of renal injury to a certain extent. This study can improve gynecologists’ understanding of pelvic anatomical variations to some degree. Combined with the findings of the LION trial and the vulnerable anatomical characteristics of pelvic ectopic kidney, if enlarged left pelvic lymph nodes are identified intraoperatively, selective lymph node sampling should be prioritized over systematic lymphadenectomy to reduce the risk of injury to the perirenal blood vessels and ureter of the ectopic kidney. Meanwhile, intraoperative clear identification of aberrant renal vessels and ureter as well as delicate anatomical dissection are required. Intraoperative urological consultation should be performed when necessary to achieve renal protection.

It is important to acknowledge the limitations of this study. First, this study only reports one case of advanced ovarian cancer combined with pelvic ectopic kidney. The extremely small sample size prevents us from evaluating the reproducibility and safety of the treatment strategy, nor can we conduct any statistical inferences. Second, the follow-up period is relatively short, resulting in a lack of long-term oncological outcomes and long-term renal function data. Third, the initial imaging examination at another hospital misdiagnosed the pelvic ectopic kidney as left renal agenesis; however, the original MRI images are unavailable. Based on our clinical experience, the initial misdiagnosis of pelvic ectopic kidney at the referring hospital was likely due to marked pelvic visceral prolapse or malrotation, which obscured the typical anatomic landmarks on routine non-contrast imaging. This study only reviews the misdiagnosis through textual description, lacking the objective presentation of key imaging features of the misdiagnosis and opportunities for readers to verify independently. Fourth, due to the rarity of this case, there is a lack of control or comparison cohorts, which could have been valuable for in-depth research. Fifth, this study cannot compare with all published literature, leading to insufficient breadth of the literature review. However, given the rarity of this specific clinical scenario, this report provides valuable insights and a practical framework for other clinicians. Future multi-center studies are needed to accumulate more data and establish evidence-based guidelines for managing such complex cases.

We emphasize that this favorable outcome was contingent upon several specific factors: preserved renal function, absent tumor invasion into the ipsilateral kidney or ureter, negative pelvic lymph nodes, and localized adhesions with identifiable dissection planes. This approach is therefore applicable only when these conditions are met. It would be contraindicated in patients with compromised renal function or solitary kidney, tumor involvement of the ectopic kidney or renal hilum, extensive dense adhesions, or enlarged suspicious lymph nodes requiring systematic lymphadenectomy near the ectopic kidney. As a single proof-of-concept case, our findings should not be generalized without further validation.

## Conclusion

This case report demonstrates that cytoreductive surgery for advanced ovarian cancer in a patient with a pelvic ectopic kidney is both safe and feasible under the specific conditions of this case. Through comprehensive preoperative analysis, it is feasible to avoid intraoperative injury to the ectopic kidney, prevent postoperative impairment of renal function, and eliminate unnecessary nephrectomy. The key to a successful outcome lies in a high index of suspicion for anatomical variants, a thorough preoperative workup using advanced imaging and functional studies, and a collaborative multidisciplinary approach. By accurately identifying and meticulously preserving the ectopic kidney, it is possible to achieve complete tumor resection without compromising renal function, thereby improving the patient’s quality of life and long-term prognosis.

## Data Availability

The original contributions presented in the study are included in the article/**Supplementary Material**. Further inquiries can be directed to the corresponding author.

## Supplementary material

Supplementary Figure 1 Schematic diagrams comparing normal renal anatomy and pelvic ectopic kidney anatomy. **(A)** Normal renal anatomy: The left renal artery arises from the lateral wall of the abdominal aorta, and the left renal vein drains into the inferior vena cava. **(B)** Pelvic ectopic kidney anatomy: The left renal artery originates aberrantly from the left common iliac artery, with anomalous courses of the left renal vein and left ureter deviating from standard renal architecture.

Supplementary Figure 2 Preoperative imaging showing no obvious tumor infiltration around the left pelvic ectopic kidney.The left pelvic ectopic kidney is clearly delineated, with no evidence of tumor invasion or infiltration in the perirenal region.

Supplementary Figure 3 Renal function remained within normal limits during neoadjuvant chemotherapy (NACT).Serum renal function parameters stayed stable throughout 4 cycles of NACT, indicating no chemotherapy‑related renal impairment.
