# Supplementary figures and images for: Cytoreductive surgery for ovarian cancer with pelvic ectopic kidney: a case report
Source: Front Oncol. 2026 May 15;16:1815298. doi: 10.3389/fonc.2026.1815298 (PMC13219304; doi:10.3389/fonc.2026.1815298)

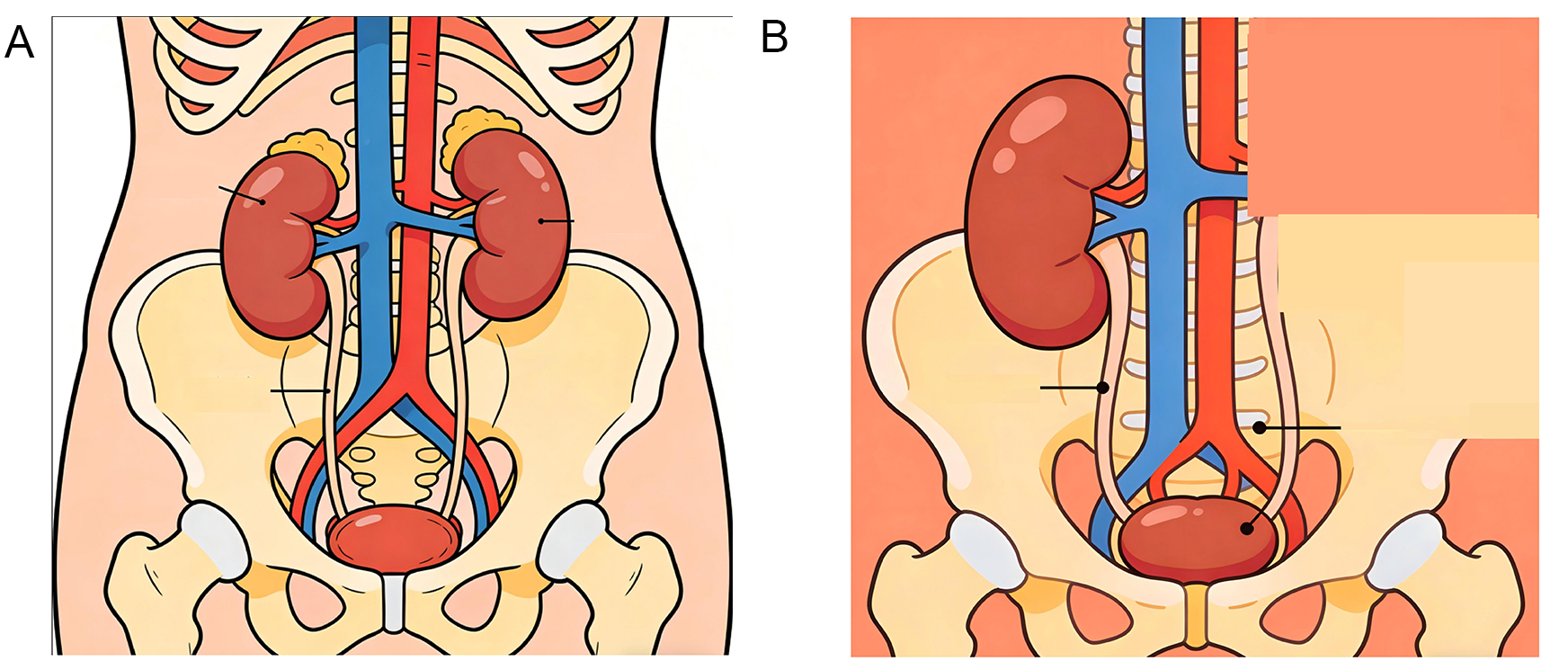

Supplement: Supplementary Figure 1 — Schematic diagrams comparing normal renal anatomy and pelvic ectopic kidney anatomy. (A) Normal renal anatomy: The left renal artery arises from the lateral wall of the abdominal aorta, and the left renal vein drains into the inferior vena cava. (B) Pelvic ectopic kidney anatomy: The left renal artery originates aberrantly from the left common iliac artery, with anomalous courses of the left renal vein and left ureter deviating from standard renal architecture. [file Image1.tif]

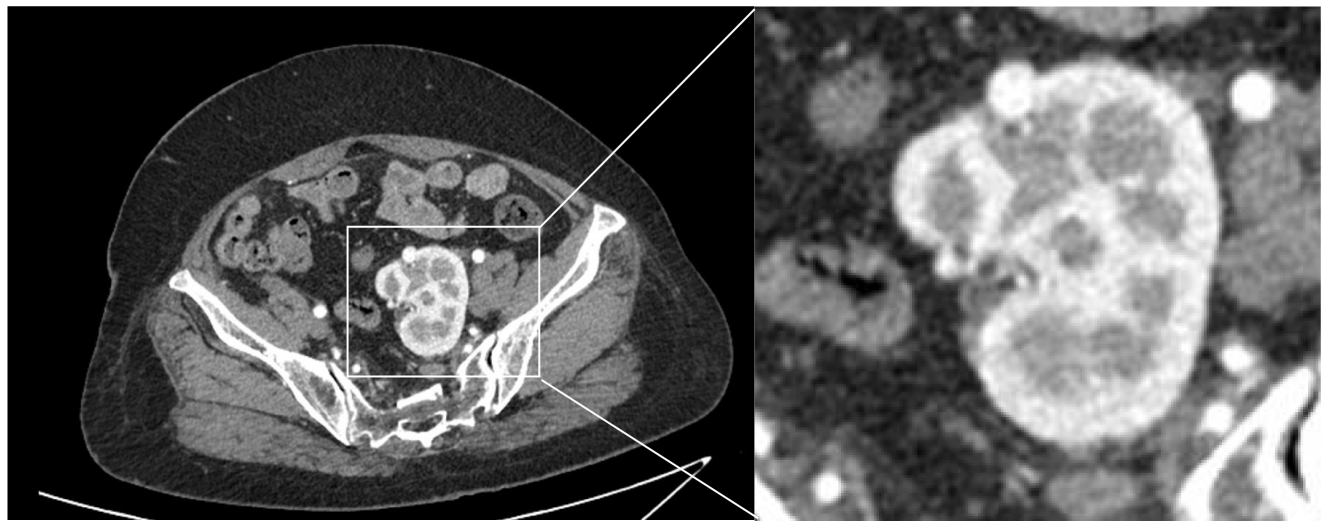

Supplement: Supplementary Figure 2 — Preoperative imaging showing no obvious tumor infiltration around the left pelvic ectopic kidney.The left pelvic ectopic kidney is clearly delineated, with no evidence of tumor invasion or infiltration in the perirenal region. [file Image2.tiff]

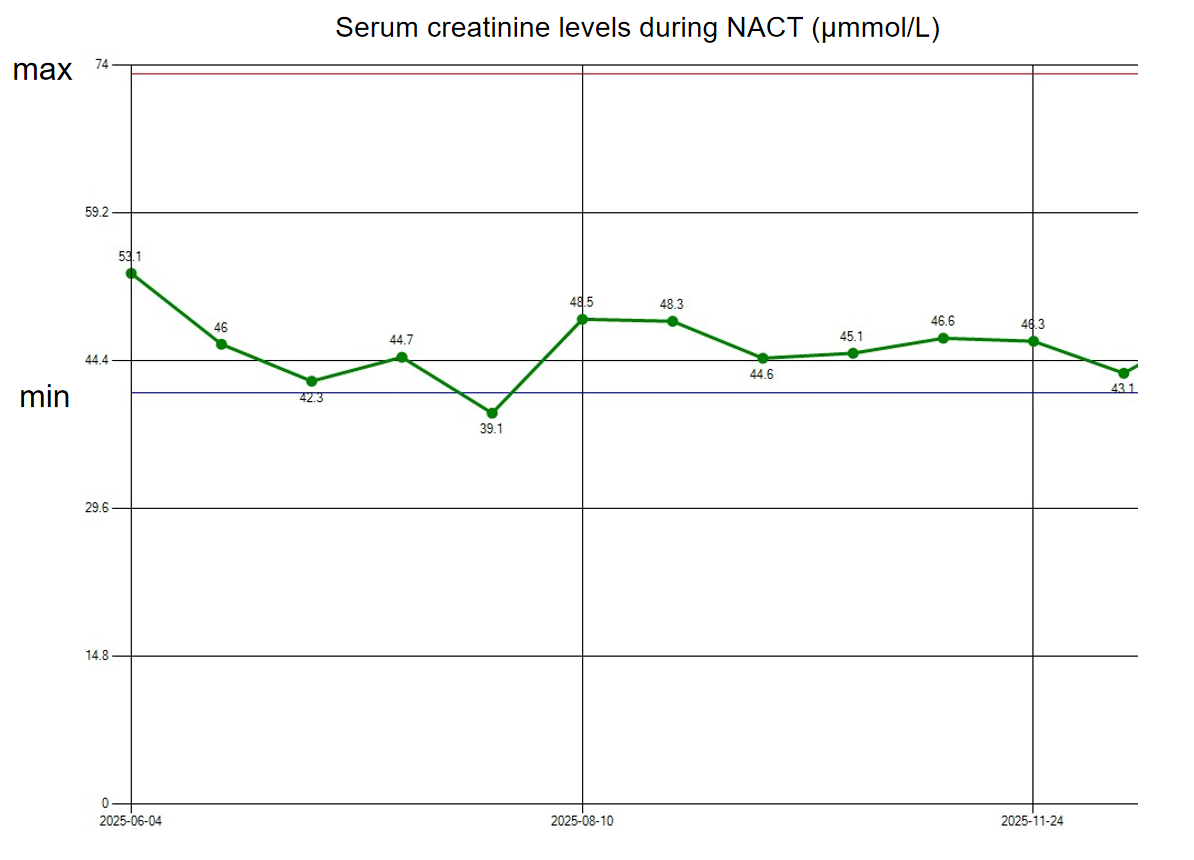

Supplement: Supplementary Figure 3 — Renal function remained within normal limits during neoadjuvant chemotherapy (NACT).Serum renal function parameters stayed stable throughout 4 cycles of NACT, indicating no chemotherapy‑related renal impairment. [file Image3.png]
